# Toughening of Epoxy Systems with Interpenetrating Polymer Network (IPN): A Review

**DOI:** 10.3390/polym12091908

**Published:** 2020-08-24

**Authors:** Ujala Farooq, Julie Teuwen, Clemens Dransfeld

**Affiliations:** Faculty of Aerospace Engineering, Aerospace Manufacturing Technologies, Delft University of Technology, Kluyverweg 1, 2629 HS Delft, The Netherlands; J.J.E.Teuwen@tudelft.nl (J.T.); C.A.Dransfeld@tudelft.nl (C.D.)

**Keywords:** IPN, semi-IPN, toughening, epoxy, morphology, mechanical properties

## Abstract

Epoxy resins are widely used for different commercial applications, particularly in the aerospace industry as matrix carbon fibre reinforced polymers composite. This is due to their excellent properties, i.e., ease of processing, low cost, superior mechanical, thermal and electrical properties. However, a pure epoxy system possesses some inherent shortcomings, such as brittleness and low elongation after cure, limiting performance of the composite. Several approaches to toughen epoxy systems have been explored, of which formation of the interpenetrating polymer network (IPN) has gained increasing attention. This methodology usually results in better mechanical properties (e.g., fracture toughness) of the modified epoxy system. Ideally, IPNs result in a synergistic combination of desirable properties of two different polymers, i.e., improved toughness comes from the toughener while thermosets are responsible for high service temperature. Three main parameters influence the mechanical response of IPN toughened systems: (i) the chemical structure of the constituents, (ii) the toughener content and finally and (iii) the type and scale of the resulting morphology. Various synthesis routes exist for the creation of IPN giving different means of control of the IPN structure and also offering different processing routes for making composites. The aim of this review is to provide an overview of the current state-of-the-art on toughening of epoxy matrix system through formation of IPN structure, either by using thermoplastics or thermosets. Moreover, the potential of IPN based epoxy systems is explored for the formation of composites particularly for aerospace applications.

## 1. Introduction

Epoxy thermosetting resins were discovered between the 1930s and 40s by P. Schlack [1] and P. Castan [2] and possess excellent features such as low cost, ease of processing, better chemical and thermal resistance, superior electrical and mechanical properties, etc. These characteristics enable its use as a potential candidate for a broad range of applications such as electrical and electronic devices, aerospace and automotive manufacturing, etc. [3,4,5]. The major drawbacks of the un-modified epoxy resins, including brittleness and low elongation after cure, restrict their application in different fields [6,7]. In epoxy resins, crack formation usually occurs on their free surfaces which spreads further with the absorption of energy, leading to fracture [6]. Thus, it is of particular interest to improve the fracture toughness of epoxy in order to unleash its full potential. Fracture toughness of a material is typically defined as its resistance against the crack growth and usually estimated in terms of stress intensity factor K_C_ or critical strain energy release rate G_C_ [8]. This limitation of brittle failure is also seen when epoxy thermosetting systems are used as matrix in carbon fibre reinforced polymer composites as high performance lightweight materials, including in the aerospace sector. The fibre-reinforced composite materials usually suffer from poor resistance to inter-laminar fracture induced by impact damage due to the lower toughness and crack resistance of epoxy matrix. Therefore, the fracture toughness of the matrix affects the damage tolerance of the composites, which is relevant in aerospace applications, and, consequently, the toughening of epoxy resin has received significant attention [9,10,11,12,13].

Different methodologies have been proposed in literature to enhance the fracture toughness of epoxies, including: (a) variation in cross-link density and molecular weight of the epoxy system itself, (b) chemical modification of the epoxy by incorporating flexible backbone and (c) integration of a second phase (i.e., rubber, thermoplastics, hyper-branched polymers, inorganic nanoparticles, etc.) [7,14,15]. However, all these approaches have limitations which need to be considered for the selection of an appropriate toughening strategy for epoxies for high-temperature applications required for aerospace structures. The first strategy of reducing the cross-link density and respectively increasing the molecular weight between crosslinks of the epoxy system usually results enhancement in fracture toughness but with significant decrease in other properties, including the glass transition temperature, tensile and flexural strength [16]. The second approach of epoxy toughening, achieved by chemically modifying its backbone and thus allowing more mobility between the cross links, may alter the thermal stability (i.e., *T*_g_) of the epoxy system. Current industrial formulated systems typically represent a good trade-off between the two mechanisms mentioned above. The third approach, the incorporation of a ductile phase (i.e., second phase) into the epoxy system can also lead to undesired results. For instance, the addition of liquid rubber such as carboxyl-terminated butadiene–acrylonitrile or amine-terminated butadiene–acrylonitrile [17,18,19,20,21] into the epoxy resin is known to increase its fracture toughness but comes with the loss of thermal stability due to the lower *T*_g_ of the added phase. Therefore, the addition of amorphous thermoplastics (i.e., polyethersulfone, polycarbonate, etc.), which undergo dissolution, followed by reaction induced phase separation, has been used as an alternative solution [22,23,24]. However, this strategy of improving fracture toughness of epoxy also poses some challenges in dissolving high molecular weight thermoplastic in epoxy resin, resulting in poor processability due to the increase in viscosity. A more recent approach to avoid these processing problems addresses the formation of IPN via in-situ polymerization of the toughener component [25].

**Figure 1:**
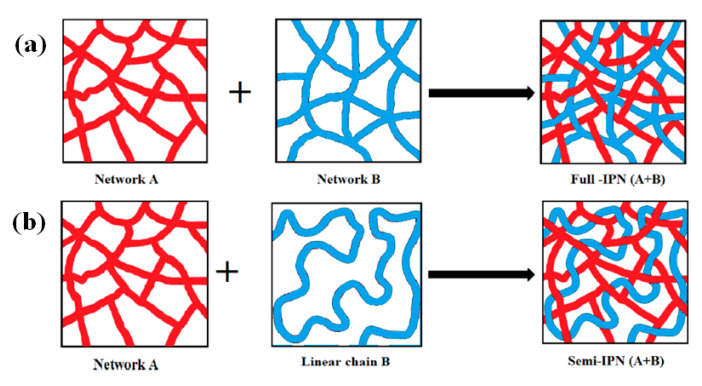
Schematics of the formation of (**a**) full interpenetrating polymer network (IPN) and (**b**) semi-IPN structures. Reprinted with permission from ref. [28], Copyright [2018], American Chemical Society.

The term “IPN” was first introduced by John Millar in 1960 [26]. An interpenetrating polymer network (IPN) is a class of polymeric system with no covalent bonds between two or more polymeric networks, which are at least partially interlocked on a molecular scale. Moreover, a complete interpenetration is not usually observed on a molecular scale in IPN while particulate or bi-continuous morphologies are rather formed with characteristic length scales in the order of tens of nanometers. Intertwined morphologies of various scales can also occur through phase separation, also referred to as spinodal decomposition. They are driven by the thermodynamic stability of a homogeneous solution based on Cahn Hilliard free energy with diffusion driven dynamics [27]. The phase behavior can be characterized by an upper critical solution temperature (UCST) or a lower critical solution temperature (LCST). In polymers we can observe both phenomena, IPN and spinodal decompositions, or a combination thereof. In literature the use of the terminology IPN is somewhat ambiguous and incoherent between disciplines, as some authors refer to it as interpenetrating polymer networks at the polymer scale, while others also refer to it as phase separation phenomena in polymers at larger scales. In an IPN structure, the network of two cross-linked polymers is interlaced with each other at the polymer scale (Figure 1a) while in semi-IPN the interlaced networked is formed by the combination of one cross-linked (thermosetting) polymer and one linear (thermoplastic) polymer (Figure 1b). IPNs can be formed by dissolving the monomer of the second polymer in the network of the first polymer, followed by cross linking the second polymer to form a network (i.e., sequential IPN) or by mixing two monomers and then cross-linking them to form a network (i.e., simultaneous IPN). Another approach to form IPN consists of two steps: (i) blending of two different polymers which are thermodynamically miscible and (ii) cross-linking of these polymers to form a network [28]. An IPN structure, which is typically formed by the interpenetration and grafting reaction, for example between PU and unsaturated polyester, is termed as graft-IPN. IPN structures can be formed by using either thermoplastics or thermosets in order to toughen the brittle epoxy resins.

The evolution of the morphology during the synthesis of processing IPN systems plays an important role in defining the final properties of the system. The IPN structures can phase separate either by nucleation & growth (NG), leading to spherical inclusions or spinodal decomposition (SD) leading to co-continuous morphologies. The coalescence of these phase is however limited due to the molecular entanglement of the crosslinked phases. In the case of an in-situ polymerization, the phase behavior continuously evolves; up to four stages of evolution have been observed [29]: (i) initially, both polymers, respective of their monomers, remain soluble with a clear transparent solution, (ii) phase separation occurs during polymerization through nucleation and growth mechanism, (iii) which can in some cases transition to formation of interconnected cylinders, hence spinodal decomposition and (iv) further polymerization results in growth of domains [30]. This two-phase morphology plays a vital role in improving the characteristics of a resultant system such as thermal stability [31] or resistance to fracture. The glass transition temperature, *T*_g_, is a distinct indicator of phase morphologies and their scales: two distinct glass transition temperatures were observed for domains of larger sizes, while smaller domains show inward shifted *T*_g_, ultimately displaying one glass transition peak when homogeneously blended at the molecular scale [32,33].

The IPN structure is known to impart better mechanical properties (e.g., fracture toughness) to the modified system. There are several other interesting characteristics of IPN, including: combination of desirable properties of two different polymers, insolubility in any solvent, etc. [34]. For example, the network formation due to the combination of two non-compatible polymers, i.e., tough thermoplastic and brittle thermoset, provides inherent advantages of each component, such as improved toughness, and comes from thermoplastics while thermosets are responsible for high service temperature [28].

The objective of this article is to provide an overview of the current state-of-the-art approaches of IPN structure for the toughening of epoxy resins and also to explore potential findings for future research. In the first part, the characteristics of semi-IPN based systems by using thermoplastic tougheners will be described in detail as a function of toughener content, etc. The second part will address details about the systems modified with thermosetting tougheners. Finally, the third section addresses IPN based epoxy systems in fibre reinforced polymer composites, particularly for aerospace applications. In the same section, a case study of poly(ether imide) also considered to toughen the epoxy resin will be discussed in greater detail. The list of abbreviations is provided in Appendix A.

## 2. IPN Based Epoxy Systems with Thermoplastic Tougheners

Toughening of epoxies with thermoplastics, studied since the early 1980s, has been considered to be an effective way to improve the toughness of epoxy resins without a significant decrease in desirable properties such as stiffness and strength, thermal stability, chemical and creep resistance, and processability [25]. Several thermoplastics have been studied to toughen epoxies such as polysulfone (PSF), poly(ether sulfone) (PES), poly(ether imide) (PEI), polyamides (PA), poly(ether ether ketone) (PEEK), poly(phthalazinone ether) [35,36,37,38]. The effect of several parameters have been investigated in literature such as (1) toughener content, (2) structure and molecular weight of toughener components, (3) type of curing agent for epoxy or (4) synthesis parameters, to study the resultant mechanical properties of the thermoplastic modified epoxy systems. In the following sections, these parameters will be discussed in detail for the combination of materials studied in literature, their preparation and main findings.

### 2.1. Effect of Toughener Content

**Figure 2:**
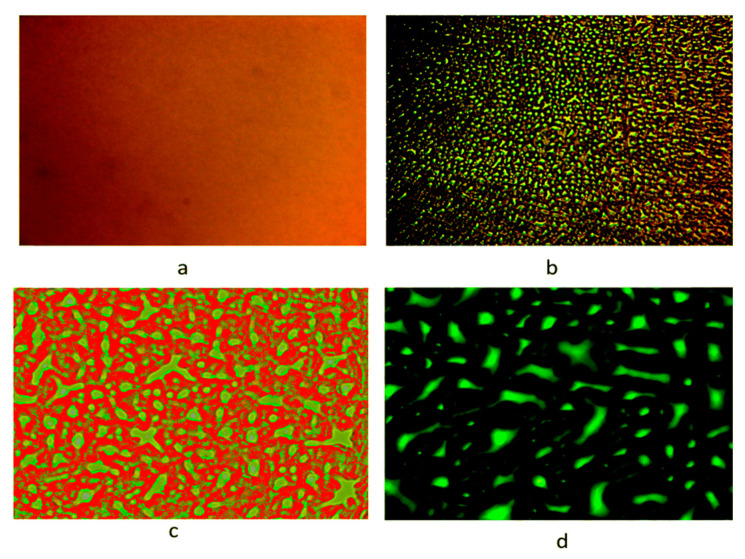
Optical micrographs of epoxy system modified with 16.6 wt % of methacrylate acrylonitrile butadiene styrene (MABS): (**a**) homogenous morphology (**b**) development of droplets of epoxy phase at 400 s, (**c**) bicontinuous structure at 550 s, and (**d**) elongated structures of the epoxy at 800 s. Reprinted with permission from ref. [39], Copyright [2019], The Royal Society of Chemistry.

Several studies have reported the effect of toughener content on the mechanical properties (e.g., fracture toughness) of modified epoxy systems. For instance, study on the incorporation of methyl methacrylate acrylonitrile butadiene styrene (MABS) copolymer into the epoxy resin (DGEBA/DDS) was reported by Joy et al. [39]. The authors investigated the influence of toughener content (4.76–16.6 wt %) on the resultant morphology and mechanical properties of the modified epoxy system. The optical micrographs of the toughened epoxy system taken during different stages of the epoxy crosslinking reaction showed the existence of phase-in-phase morphology (i.e., epoxy particles bounded by thermoplastic domains, which further enclosed in a continuous phase of epoxy, see Figure 2) at 16.6 wt % of toughener. Further results showed an increase in tensile strength and impact strength of about 25% and 72%, respectively, by adding 9 wt % of MABS into the epoxy; however, there was a decrease of those properties with further addition of toughener. This decrease in mechanical properties was attributed to the formation of phase-in-phase morphology, as explained above.

**Figure 3:**
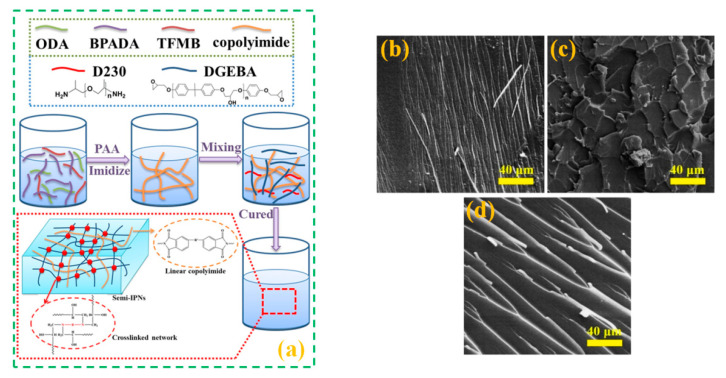
(**a**) Schematics of the synthesis procedure and structure of PI/EP semi-IPN. Reprinted with permission from ref. [40], Copyright [2019], John Wiley and Sons; SEM images of fracture surfaces of (**b**) Diels–Alder (DA) polymer, (**c**) semi-IPN sample (DA/EP 50:50), and (**d**) the epoxy sample. Reprinted with permission from ref. [41], Copyright [2019], John Wiley and Sons.

Qu et al. [40] reported the synthesis and characterization of semi-IPN using epoxy and co-polyimide. In short, the synthesis procedure started with the preparation of polyamic acid, followed by its thermal imidization. The prepared co-polyimide was mixed with epoxy oligomers (DGEBA and D-230) and then cured to form the cross-linked network (see Figure 3a). The results revealed the improvement in the mechanical properties and thermal stability of modified epoxy system as a function of increasing toughener content.

Polyethersulfone (PES) has also been used as a toughener in triglycidyl-p-aminophenol (TGAP) epoxy resin to increase its mechanical properties. The fracture toughness measured at room temperature of the PES/epoxy system was increased up to 11%, which was associated with the formation of semi-IPN structure between epoxy and PES [36]. The fracture toughness of the epoxy resin was also enhanced by adding Diels–Alder (DA) polymer into the epoxy system based on DGEBA/D230 [41]. The morphological analysis of the fractured surfaces revealed the existence of rough and irregular surfaces for the semi-IPN based sample, as compared to the samples without toughener (Figure 3b–d), which was attributed to the higher work of fracture due to the presence of semi-IPN structure.

Liu et al. [42] reported the synthesis of semi-IPN by blending epoxy (BADCy) and thermoplastic polyimide (TPI) without any addition of solvent. The prepared blends displayed improved processability, as compared to the neat epoxy, as shown by the reduction in gelation time at 200 °C and lower values of viscosity onset temperature. The SEM analysis revealed the formation of phase-separated morphology along with the continuous phases of TPI, at higher concentration of toughener (≥15%). The incorporation of TPI in epoxy resin resulted in better mechanical (i.e., fracture toughness, impact strength, tensile strength, etc.) and dielectric properties without losing its heat resistance (i.e., *T*_g_).

Wang et al. [10] reported the synthesis of a toughening agent based on a hyper-branched polyester having flexible chain blocking. By incorporating 20 wt % of toughener in epoxy phase, an increase of 122% and 184% was observed in bending strength and impact strength, respectively. This increase was linked to the formation of IPN between flexible chain of toughener and epoxy molecular chain (i.e., cavity toughening mechanism). The toughening of epoxy resin was also reported by the presence of polysulfide fraction resulted from the UV-induced polymerization of the thiol-ene/epoxy system [43]. The results revealed the enhancement in the mechanical properties (i.e., impact strength and fracture toughness) of epoxy system as a function of increasing content of polysulfide due to its flexible nature.

**Figure 4:**
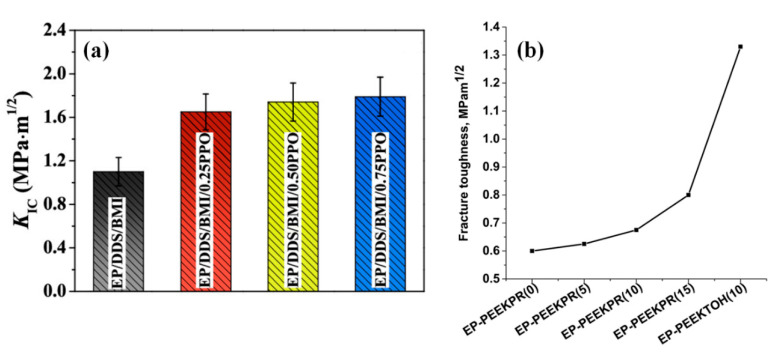
(**a**) Fracture toughness of neat epoxy and epoxy system modified with different content of polyphenylene ether (PPO). Reprinted with permission from ref. [44], Copyright [2018], American Chemical Society, (**b**) fracture toughness of epoxy blends with PEEK-PR or PEEK-TOH. Reprinted with permission from ref. [38], Copyright [2019], John Wiley and Sons.

The simultaneous IPN approach has been reported to produce in-situ nanofibres of polyphenylene ether (PPO) by polymerization force during the cure phase of thermosetting epoxy resin [44]. The epoxy modified with PPO displayed 63% higher fracture toughness and 30% higher tensile strength as compared to the neat system at room temperature, which was attributed to the reinforcing and toughening functions of aligned PPO fibres, in addition to the IPN. The fracture toughness measured at room temperature increased (from 50% to 63%) by increasing the content of PPO from 10 to 25 wt % (Figure 4a) [44]. Similarly, an increasing behavior of fracture toughness as a function of toughener content (i.e., PEEK-PR) was also observed, with the highest value of 33% increase in fracture toughness [38]. However, the highest fracture toughness (1.33 MPa·m^1/2^) was observed by adding PEEK-TOH in the epoxy system, which was associated with its improved interaction with the epoxy resin via polar hydroxyl groups (Figure 4b) [38]. Likewise, polymethyl methracrylate (PMMA) and polycarbonate (PC) were reported to use as toughening agents for epoxy resin [45]. The results showed better mechanical properties, i.e., tensile, flexural, and impact strength for epoxy system toughened with 4 wt % of PMMA or 6 wt % of PC. Moreover, PMMA toughened epoxy/silk composites exhibited higher elongation at break (4.39%) as compared to the pure epoxy resin (3.98%) and PC toughened epoxy/silk composites (3.68%) [45].

Bhuniya and Adhikari [11] investigated the toughening of epoxy resin using hydroxy-terminated silicon-modified polyurethane (SiMPU) oligomers, prepared from dimethyl dichlorosilane, poly(ethylene glycol) and toluene 2,4-Diisocyanate. The impact strength and fracture toughness of modified epoxy system increased gradually till 16.7 wt % of SiMPU, with highest values of 25 J/m and 3 MPa·m^1/2^, respectively, at this concentration (i.e., 16.7 wt % SiMPU). Above this content of SiMPU, the enhancement in the fracture toughness and impact strength of epoxy system was negligible [11]. Moreover, different types of nonisocyanate polyurethanes have also been reported to toughen the epoxy resin through the formation of IPN structure, hydrogen bonding and urethane linkage [46,47].

**Figure 5:**
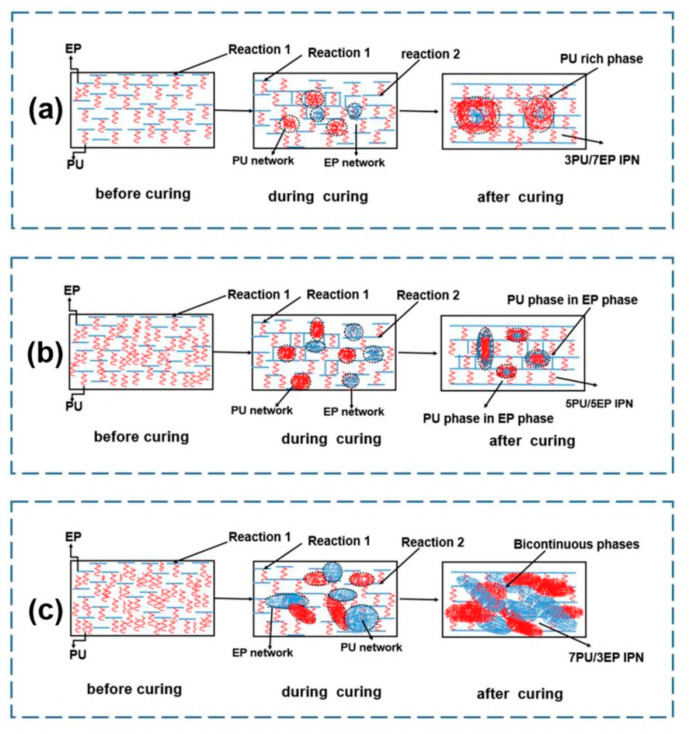
Schematic representation of structural development in epoxy/PU systems with (**a**) 30 wt % PU, (**b**) 50 wt % PU and (**c**) 70 wt % PU. Reaction 1: grafting reaction between -NCO groups of PU and side -OH groups of epoxy (before adding curing agent); Reaction 2: reaction between -NCO groups of PU network and -OH groups of epoxy network (after adding curing agent). Reprinted with permission from ref. [50], Copyright [2019], Elsevier.

Polyurethane (PU) is typically prepared by the reaction of a polyol with a diisocyanate, with or without the addition of catalyst. PU chains can have a high degree of mobility and can therefore contribute to energy dissipation during fracture. Several studies have reported the modification of epoxy system using PU via graft-IPN mechanisms [48,49]. In a study aiming to improve tribological properties, the toughening of epoxy resin (DGEBA/MOCA) was also performed by creating graft-IPN using PU [50]. A graft IPN architecture was created by reaction of the isocyanate group of the PU with the hydroxy group of the DGEBA (left image of Figure 5a). After adding the MOCA curing agent, the network started to form through epoxy-amine reactions, leading to distinct phase separation morphologies with PU rich and epoxy rich domains, depending on the PU content in the system. Incorporating PU (30 wt %) into the epoxy phase without curing agent (MOCA), led to phase separation between PU and epoxy by reacting with curing agent and PU networks also interpenetrated into the epoxy networks (middle image of Figure 5a). Furthermore, gelation of PU phase resulted in the formation of PU vesicles, which in turn entrapped epoxy domains (right image of Figure 5a). By further increasing the PU content (50 wt %), phase-in-phase morphology (entrapment of PU phase in epoxy phase) was observed (right image of Figure 5b). Finally, a bi-continuous structure was obtained at 70 wt % of PU, where PU and epoxy domains interpenetrated each other. This behavior was attributed to the enhanced viscoelastic phase separation caused by the large size disparity between PU and epoxy (right image of Figure 5c) [50]. The best tensile strength of modified epoxy was obtained by adding 30 wt % of polyurethane prepolymer (PUP) and decreased by further addition of PUP. The impact strength, on the other hand, increased by increasing PUP content. The wear performance of epoxy systems with 30 and 50 wt % of PUP was better than the next epoxy and PUP [50].

Similarly, the modified epoxy system prepared by the graft copolymerization between PU and commercial epoxy resin (E-44) has also been reported in literature [51]. The soft PU phase was synthesized from 2,4-toluene diisocyanate trimer (TDIT) and poly(propylene glycol) (PPG). The results showed an increase in impact and compressive strength as a function of increasing PUP content up to 16.7 wt % and a decrease afterwards. At 16.7 wt % of PUP, the compressive strength reached 184.8 MPa and the impact strength 76.6 kJ/m^2^, which was attributed to the flexible segments of toughener and also to the formation of graft-IPN structures [51]. An overview of different IPN based systems is presented in Table 1, in terms of toughener content, morphology, investigated properties and key findings.

### 2.2. Effect of Other Parameters

Apart from toughener content, other parameters on the resultant properties of the modified epoxy system has also been investigated in literature. These parameters include type of curing agent, structure and molecular weight of the components, synthesis parameters, etc. 

**Figure 6:**
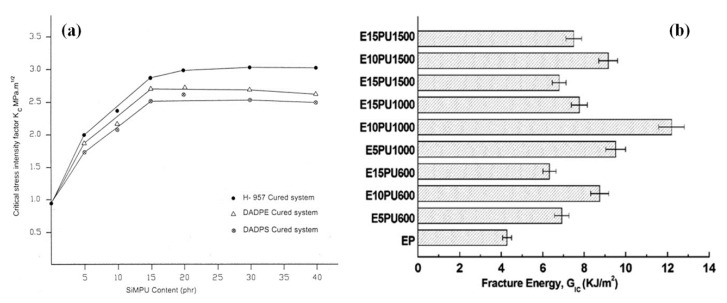
(**a**) Influence of different curing agents (H-957, DADPE and DADPS) on the stress intensity factor, K_C_ of modified epoxy systems. Reprinted with permission from ref. [11], Copyright [2003], John Wiley and Sons. (**b**) Fracture energy of PU modified epoxy systems obtained with various molecular weights (600–1500 g·mol^−1^) of polyethylene glycol. Reprinted with permission from ref. [57], Copyright [2014], John Wiley and Sons.

For instance, Zhang et al. [55] reported the synthesis of toughened epoxy resin for steel deck pavements by introducing a flexible chain toughener into the resin via simultaneous IPN approach. The authors compared the toughening performance of two different undisclosed curing agents (G1 and G2) having different curing rates. The results showed that the mixtures having G1 and G2 curing agent satisfied the technical index requirements of steel deck pavement. Moreover, the fatigue life of epoxy mixture cured with G1 was superior to the epoxy asphalt mixture [55]. Likewise, the mechanical properties (i.e., fracture toughness, impact strength, etc.) of modified epoxy system was, in another study, also investigated as a function of different curing agents (i.e., H-957, DADPE and DADPS) [11]. The values of impact strength and fracture toughness of epoxy system, cured by using H-957, were higher than the epoxy systems cured with other two hardeners (DADPE and DADPS) (Figure 6a). The results showed an increase of 216% in fracture toughness at room temperature by curing the epoxy with H-957 along with 16.7 wt % of SiMPU, as compared to the neat epoxy [11]. 

The synthesis of two grades of epoxy/PU IPN structures has also been reported in literature by using poly(tetramethylene ether) glycol (PTMG) of two different molecular weights (650 and 1400 g·mol^−1^) [56]. The quasi-static fracture toughness of modified epoxy, obtained from high molecular weight PTMG, showed higher values (i.e., 2.95 MPa·m^1/2^) than the system having low molecular weight PTMG (2.8 MPa·m^1/2^). However, the dynamic fracture toughness was higher (i.e., 3.1 MPa·m^1/2^) in the case of low molecular weight PTMG, as compared to the values (i.e., 2.5 MPa·m^1/2^) for epoxy system modified using high molecular weight PTMG [56]. Likewise, Chaudhary et al. [57] reported the toughening of epoxy resin using polyester polyurethane obtained from the glycolysis of PET waste. The authors investigated the toughening performance of PU by using polyethylene glycols of different molecular weights (600–1500 g·mol^−1^). The results showed the maximum increase of 184% in energy to break, 61% in tensile strength and of 212% in impact strength, respectively, by adding 10 wt % of polyurethane prepared with the glycol having molecular weight of 1000 g·mol^−1^. However, a further increase in molecular weight of glycol (i.e., 1500 g·mol^−1^) resulted in lower mechanical properties (i.e., energy to break) of the modified epoxy system. (see Figure 6b) [57].

In addition to the molecular weight of components, the effect of isocyanate index (I_nco_) being slightly over- or under-stoichiometric, 0.95 resp. 1.03, of polyurethane prepolymer on the mechanical properties of modified epoxy systems has also been investigated [58]. The better fracture properties of the modified epoxy system was achieved by using toughener with PUP having a higher I_nco_, hence being terminated with a isocyanate group (opposed to a hydroxyl group termination for a low I_nco_). The impact strength was maximally enhanced by adding 20% of polyurethane with highest isocyanate index while the addition of 15% of the toughener was enough to obtain the best tensile strength and elongation at break [58]. This was attributed to the PUP isocyanate end groups forming additional cross links with hydroxyl group from the epoxy network. In another study, the authors examined the characteristics of the modified epoxy system (epoxy/PU) synthesize under different accelerations of gravity (0, 1 and 2 g) [59]. The bending stress and coefficient of thermal expansion (CTE) of modified epoxy showed a decrease with the increasing simulated gravity, which was attributed to the increase in diameter and decease in number of dispersed phase [59].

**Figure 7:**
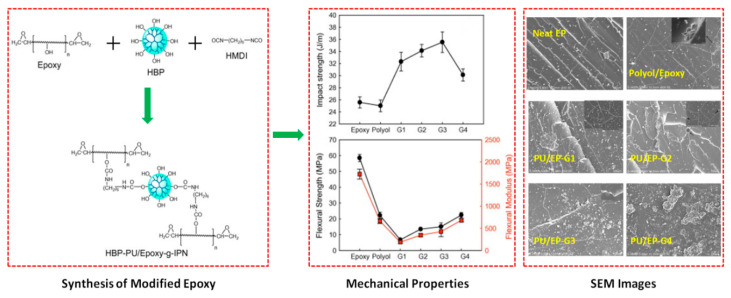
Schematics of the formation of modified epoxy system using HBPs; Impact strength, flexural strength and flexural modulus of pure epoxy, linear polyol base epoxy and modified epoxy samples having different generations of HBPs (HBP-G1 to HBP-G4); SEM images of pure epoxy, linear polyol base epoxy and modified epoxy samples having different generations of HBPs; Reprinted with permission from ref. [13], Copyright [2013], Elsevier.

Dhevi et al. [13] reported an interesting approach to toughen the epoxy resin by using hyper-branched polymers (HBPs). The authors further investigated the influence of different generations (1–4) of HBPs on the resultant characteristics of modified epoxy system. The HBPs were synthesized by using dipentaerythritol and dimethylol propionic acid via single-step melt polycondensation approach. The calculated amount of epoxy, HBP and HMDI were mixed together and cured under particular conditions, in order to obtain the final system for analysis (Figure 7). Linear polyol was also prepared using the same methodology, in order to perform the comparative analysis with HBPs. The outcome of this study showed an increase in the impact strength of modified epoxy till generation 3 and a decrease for generation 4, which was attributed to the formation of bigger particles. However, the flexural modulus and strength linearly increased as a function of generation because of the enhancement in the rigidity of the system (Figure 7). The HBP-PU based epoxy samples displayed higher toughness at room temperature as compared to the next epoxy and linear-PU based epoxy samples. This enhancement was associated to the formation of two-phase morphology during reaction and also due to the tearing of toughener particles during fracture, which in turn decreases the rate of crack growth, as also verified by the SEM images (Figure 7). However, the thermal stability and flexural characteristics of HBP-PU based epoxy system were lower than the neat epoxy which was linked with the presence of flexible linkages and reduction in cross-linked density of epoxy [13]. 

**Figure 8:**
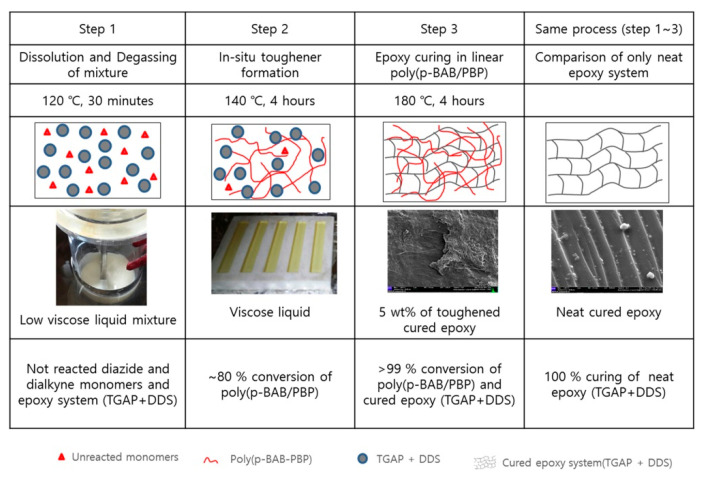
Schematics of the synthesis procedure and semi-IPN structure of neat epoxy and modified epoxy system having 5 wt % of poly(p-BAB/PBP). Reprinted with permission from ref. [60], Copyright [2019], John Wiley and Sons.

Furthermore, two novel polysulfone-based toughening agents (poly(p-BAB/SPB) and poly(m-BAB/SPB)) have been prepared through azide–alkyne polymerization reaction [35]. The in-situ toughening of epoxy resin using poly(p-BAB/SPB) displayed higher fracture toughness, lower viscosity (i.e., improved processability) and better thermal resistance. The results showed that only 5 wt % of poly(p-BAB/SPB) was required to obtain the fracture toughness higher than the toughness of epoxy system modified with 20 wt % of PES at room temperature [35]. The same research group also reported the synthesis of poly(ether ether ketone) based toughening agent, poly(p-BAB/PBP), for epoxy modification [60]. The preparation process and structure of neat and poly(p-BAB/PBP) modified epoxy systems is shown in Figure 8. The fracture toughness of epoxy system modified with 5 wt % of poly(p-BAB/PBP) was even higher than the system containing the same amount of poly(p-BAB/SPB) (i.e., sulfone based toughener). The summary of different IPN based systems, investigated in literature to analyze the influence of different parameters on the properties of epoxy system, is presented in Table 2.

In addition to the above mentioned parameters, curing temperature can also affect the mechanical properties (i.e., fracture toughness) of the toughened epoxy resin. For instance, exploiting the phase behavior, Mimura et al. [61] investigated the influence of two different curing temperatures (140 and 180 °C) on the fracture toughness of epoxy/PES system. As the mixture of epoxy with PES exhibits a lower critical solution temperature, the results showed that the epoxy resin, with 10 wt % PES, cured at 140 °C below the phase separation boundary exhibited a homogeneous phase morphology, while curing at 180 °C lead to a heterogeneous phase separated morphology in the micron scale. Interestingly the homogeneous morpohology showed a single *T*_g_ while the phase separated one and the two *T*_g_ of its constituents. The Fracture toughness of the homogeneous morphology was higher at low PES contents (up to10%) and become more effective in the phase separated case for higher PES contents only. The processing viscosity of the uncured systems increases a lot due to the high molecular weight of the PES.

In summary, the effect of several parameters on the mechanical properties (i.e., fracture toughness and impact strength) of the toughened epoxy system has been investigated in literature. The increase in toughener content typically lead towards the gradual increase in fracture toughness of epoxy resin, which is attributed to the formation of IPN structure and phase separated morphologies [23,38,44]. However, beyond a certain concentration of toughener, the fracture toughness of modified epoxy system tends to reach a plateau, which is associated to the fact that there is no further interactions between the epoxy and toughener [11]. Likewise, the impact strength and tensile strength also observed to increase as a function of increasing toughener content up to a certain level and then decrease with further addition of toughener. This particular behavior is usually linked with the formation of toughener rich domains, which can inhibit the development of IPN structure [50,51]. Therefore, the toughener content is a crucial parameter to control the mechanical characteristics, i.e., fracture toughness, of the modified epoxy system. Moreover, in addition to the toughener content, other parameters have also been investigated in literature as controlling parameters of the mechanical characteristics of toughened epoxy resin such as molecular weight of toughener components, different curing agents, or curing temperatures. For example, the higher fracture toughness (at room temperature)of epoxy system is found by using toughener (PU) having higher isocyanate terminated PUPs, which is due to the higher interpenetration of additional crosslinks into the rigid epoxy matrix [58]. The molecular weight of the components, used to prepare the toughener, can also significantly affect the resultant mechanical properties (i.e., fracture toughness) of the epoxy system [56,57]. Hence, the final mechanical properties including fracture toughness of the modified epoxy system can be finely tuned by controlling the morphology and/or creating the IPN structure. Also, specific curing cycles may be used to promote or suppress phase separation in systems having an upper or lower critical solution temperatures.

## 3. IPN Based Epoxy Systems with Thermosetting Tougheners 

As seen in the previous chapter, the incorporation of a ductile thermoplastic phase in a brittle epoxy resin usually results in a viscosity increment, which in turn affects the process ability of the epoxy system [61]. Furthermore, other modification approaches such as varying the molecular weight of resin [62], reducing its crosslinked density or chemically modifying the backbone of epoxy resin also provide limited enhancement in the toughness of epoxy resin [63]. Therefore, an alternative approach is to incorporate a thermoset phase instead of a thermoplastic, in order to toughen the epoxy system.

**Figure 9:**
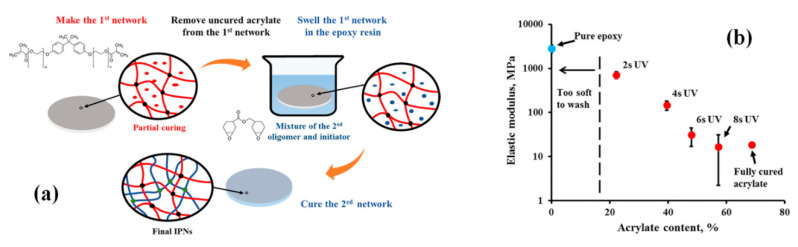
(**a**) Schematic representation of synthesis procedure of sequential acrylate/epoxy IPN, (**b**) elastic modulus as a function of acrylate content for sequential acrylate/epoxy IPN. Reprinted with permission from ref. [65], Copyright [2019], John Wiley and Sons.

For example, Fu et al. [64] reported the toughening of epoxy resin (DGEBA/DDM) using hyper-branched epoxy resin (i.e., HTTE), as a function of toughener content and generation of hyper-branched epoxy. The morphological analysis revealed the existence of cavitations in the center and fibrous yielding at the edges, which suggested the following mechanisms of toughening: particle cavitation, shear yielding of matrix and in-situ toughening. In this study, the fracture toughness was observed to increase with the increasing content of HTTE. The thermal stability of hybrid epoxy decreased with the increasing HTTE content but increased by changing the generation of HTTE from 1 to 3. This increase in thermal stability of hybrid epoxy was linked with the increase in molar mass between crosslinks, intermolecular interactions of HTTE/epoxy and crosslinked density [64]. Similarly, acrylate based epoxy system was used to toughen the epoxy resin (ECH) by varying the toughener content and the duration of UV exposure for curing [65]. The sequential IPN approach was used to prepare the modified epoxy system, i.e., the acrylate resin was partially photo-polymerized and then swollen by the epoxy resin followed by the complete curing to make IPN (Figure 9a). The nano-mechanical analysis showed that the elastic modulus of modified epoxy resin decreased from 715 to 16.7 MPa by increasing both acrylate content and UV exposure during curing (Figure 9b) [65].

Wen et al. [66] investigated the thermal and mechanical characteristics of IPN structure based epoxy systems prepared by using fluorinated ethynyl-terminated imide (FETI) oligomers and epoxy resin (BADCy) via solvent-free approach. The superior thermal and mechanical properties of blended epoxy systems were observed with the *T*_g_ of 303–312 °C, tensile strength of 87–95 MPa and impact strength of 27–37 kJ·m^−2^. These enhancements were attributed to the presence of high fluorine content, low crosslink density, high chain rigidity, and strong intra- and intermolecular interactions. This study provides some useful knowledge about developing modified epoxy resins having higher toughness without sacrificing their excellent mechanical and thermal features [66]. Similarly, the mechanical properties of epoxy resin has been enhanced by using unsaturated polyester as a thermosetting toughening agent [4].

### The Toughening of Bio-Based Epoxy (BE) Resin Has Been Performed by Using Epoxidized Soybean 

oil (ESO) and poly(furfuryl alcohol) (PFA) [67]. SEM images showed a significant change from brittle fracture surfaces, observed for neat epoxy, to the surfaces having plastic deformation in BE/ESO/PFA systems, which was attributed to the reaction induced phase separation phenomenon in blended systems. The results showed that the incorporation of ESO and PFA in epoxy system resulted in 76.6% higher impact strength, as compared to the neat epoxy, which was associated to the addition of flexible ESO chains. However, the tensile strength and glass transition temperature decreased by adding ESO and PFA in epoxy [67].

Wu et al. [63] reported an interesting study on the toughening of epoxy resin by adopting three approaches in a single system: (i) incorporation of tri-functional acrylate into the epoxy system to reduce its viscosity and also to form the sequential IPN, (ii) addition of scrap leather fibres (SLFs) to toughen the epoxy resin, and (iii) addition of functionalized montmorillonite (OMMTs), in order to further enhance the mechanical properties of epoxy/SLFs systems. The mechanical and thermal properties of epoxy/acrylate blend were enhanced by adding the fillers (OMMTs and SLFs). The optimum toughening-strengthening properties were obtained by incorporating 0.5 wt % of SLFs and 4.8 wt % of OMMTs in epoxy blend [63]. 

In conclusion, apart from toughening of epoxy resin with thermoplastics, different thermosetting polymeric systems have also been examined to toughen epoxies. The incorporation of thermosetting toughener usually results in enhanced fracture toughness and impact strength of epoxy system [66,68], which further shows an increasing trend as a function of increasing toughener content [64,69,70]. However, the tensile strength and thermal stability of the epoxy resin is observed to decrease by adding thermosetting toughener [64,67]. This decrease in thermal stability of epoxy system can be tackled by playing with the branching and structure of the thermosetting toughener, as evident by the increase in thermal properties of the modified epoxy as a function of varying generations of hyper-branched thermosetting toughener [64]. The summary of different IPN based epoxy systems, modified by using thermosets, is presented in Table 3.

## 4. IPN in Aerospace Applications

The enhancement in the damage tolerance of epoxy matrices of fibre reinforced composites has become a serious concern particularly for the aerospace industry, and consequently, toughening of epoxy resin has received significant attention. As already discussed, the formation of (semi-)IPN structures can lead to the systems having higher fracture toughness and other mechanical properties i.e., elongation at break, tensile strength and modulus. Furthermore, this increase in mechanical properties can further be tuned by playing with different parameters such as toughener content, structure and molecular weight of toughener components, curing agent, synthesis parameters, etc. Therefore, in this section, this enhancement in fracture toughness of epoxy-based fibre-reinforced composites commonly used for aerospace applications by forming (semi-)IPN structure will be discussed. Furthermore, the change in morphology by incorporating fibre into toughened epoxy will be explained.

Likewise, glass fibre reinforced composites were prepared by using a mixture of PEN and BA-ph as a matrix [72]. The results displayed that the curing reaction of BA-ph was hindered by the existence of PEN. However, the mechanical characteristics of the prepared composites were enhanced as a function of PEN content, which was attributed to the improvement in the interfacial adhesion and also to the formation of semi-IPN structure [72]. The mechanical characteristics (e.g., compressive strength, flexural strength and fracture toughness) of polymeric composites based on PMSQ resin and short silica fibre has also been investigated in literature as a function of the incorporation of phenolic polymer as a toughener [73]. The outcome of the study verified that the addition of toughener resulted in enhanced fracture toughness of PMSQ-silica fibre composites, with the optimum properties at 25% of toughener. This enhancement in the properties was linked to the formation of IPN, complete miscibility of IPN structure and its improved interaction with the silica fibres [73]. Similar enhancement in damage tolerance of novel aerospace grade thin-ply composite system was observed by toughening the epoxy resin [74]. The mechanical properties (e.g., fracture toughness) and phase morphology of toughened polymer composites are also dependent on heating/curing rate and surface properties of fibre reinforcement, in addition to the toughener type and content [75,76]. 

The efficient joining of different parts, in order to prepare a complete component, is very crucial for aerospace applications. Therefore, the mechanical properties of adhesives, used to join steel and fibre-reinforced polymeric components, have also been modified by using the approach of IPN structure. The authors reported the formation of micro-phase separated IPN using DGEBA/PDCPD and investigated their adhesive performance as compared to the neat epoxy adhesive [69]. The blended epoxy adhesive system displayed better mechanical properties (i.e., fracture toughness, bond strength, tensile strength, tensile modulus, etc.) than the individual components. The results showed an increase in energy dissipation mechanisms (i.e., fracture toughness) by increasing PDCPD content in epoxy. The epoxy system having 70% of PDCPD exhibited bond strength of ~35% and ~125% higher than the neat epoxy and neat PDCPD, respectively [69]. 

### Case Study: PEI as a Thermoplastics Toughener

Polyetherimide (PEI) belongs to the class of amorphous engineering thermoplastic and has an interesting set of properties including high glass transition temperature of 217 °C, higher strength and rigidity at elevated temperatures, lower shrinkage, intrinsic flame retardancy, higher moisture resistance, higher dielectric resistivity, lower coefficient of thermal expansion, etc. [77]. Moreover, PEI has good compatibility and miscibility with epoxy resins, which makes it a potential candidate for the toughening of epoxy systems. 

The fracture toughness of high performance epoxy resin can be enhanced by incorporating PEI or a modified form of PEI. Curing of an epoxy resin in the presence of PEI, can lead to local dissolution of the PEI in the epoxy, followed by a reaction-induced phase separation. This inter-diffusion process results in the formation of semi-IPN structure at the epoxy-PEI interface. The diffusion of PEI into the epoxy resin increases its plasticity and also induces stress relaxation via local shear deformation. The increment in toughness is mainly controlled by the phase separation between PEI and epoxy during curing. Moreover, the fracture energy is dissipated by the ductile deformation of dispersed PEI phase, which occurs by the stress transfer through semi-IPN structure [78].

**Figure 10:**
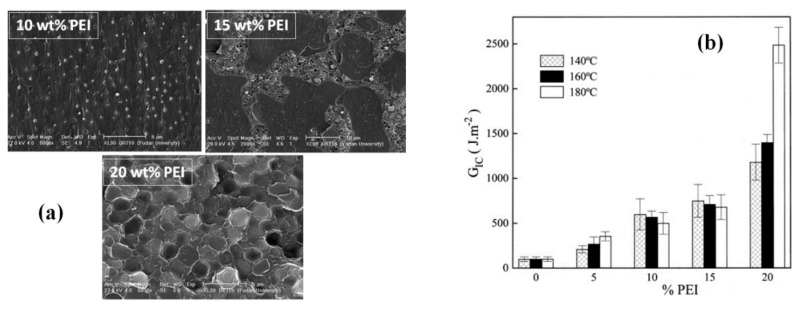
(**a**) Phase morphologies as a function of PEI content in epoxy/PEI system (particulate morphology at 10 wt % PEI, co-continuous morphology at 15 wt % PEI and phase-inverted morphology at 20 wt % PEI). Reprinted with permission from ref. [28], Copyright [2018], American Chemical Society. (**b**) Fracture energy of epoxy/PEI systems as a function of PEI content at different curing temperatures. Reprinted with permission from ref. [33], Copyright [2001], John Wiley and Sons.

The effect of PEI content on the phase morphology and mechanical properties of toughened epoxy system has been investigated in literature [30,33,79,80]. At 5 wt % of PEI in cyanate-rich epoxy, particulate morphology with very fine spherical PEI particles were observed. Similar morphology but with bigger spherical domains (d = 0.5 mm) was evident for 10 wt % PEI (Figure 10a). The proposed mechanism for this phase separation was nucleation and growth, i.e., nucleation and growth of PEI phase in epoxy matrix during curing [33]. The incorporation of 15 wt % of PEI resulted in co-continuous morphology along with the small, dispersed droplets of PEI and epoxy in epoxy-rich and PEI-rich domains, respectively (Figure 10a). The existence of these smaller dispersed droplets was associated to the secondary phase separation phenomenon in already phase separated domains [33]. A complete phase inversion was observed by increasing the PEI content up to 20 wt %, with spherical domains (d = 2 mm) of epoxy dispersed in PEI (Figure 10a). The suggested phase separation mechanism, in case of 15 and 20 wt %, was spinodal decomposition [81].

The analysis of mechanical properties of epoxy/PEI system showed that maximum toughening was obtained with 20 wt % of PEI (Figure 10b), which was attributed to the ductile deformation of PEI phase [30,33]. However, the addition of 10 wt % PEI (i.e., particulate morphology) also resulted in 2-fold increase in fracture toughness at room temperature as compared to the neat epoxy, which was linked to the crack path deflection mechanism due to PEI particles [33]. Further results displayed that the maximum tensile modulus, tensile strength and elongation at break were achieved by incorporating 15 wt % of PEI into the epoxy resin [79,80]. Mechanical properties (i.e., elongation at break (%), fracture toughness, etc.) of different epoxy/PEI systems are summarized in Table 4.

In addition to the toughening of epoxy resin, toughened polymeric composites have also been reported in literature. For instance, Turmel & Partridge [37] prepared and characterized the uni-directional fibre composite based on epoxy resin modified with PEI. During the curing phase, a distinct epoxy-rich layer was observed around the glass fibres due to the preferential wetting of the fibre by epoxy resin and hence reduced nucleation. This epoxy-rich layer was further observed to vary as a function of PEI content and the proximity of other fibres. The existence of this epoxy-rich layer in fibre composite eventually resulted in premature failure upon loading, and therefore reduced the benefit of thermoplastic toughening in terms of fracture toughness for polymer composites. The thermoplastic modification approach has also been utilized for joining different composite parts through welding. For example, carbon/epoxy and carbon/PEEK composite parts were welded together with the help of a thin PEI film [85]. The PEI layer was first co-cured on the carbon/epoxy composite part, and during curing phase, semi-IPN structure was formed due to the diffusion of epoxy resin. Subsequently, carbon/PEEK composite part was joined to the PEI film by exploiting the total miscibility between PEEK and PEI.

**Figure 11:**
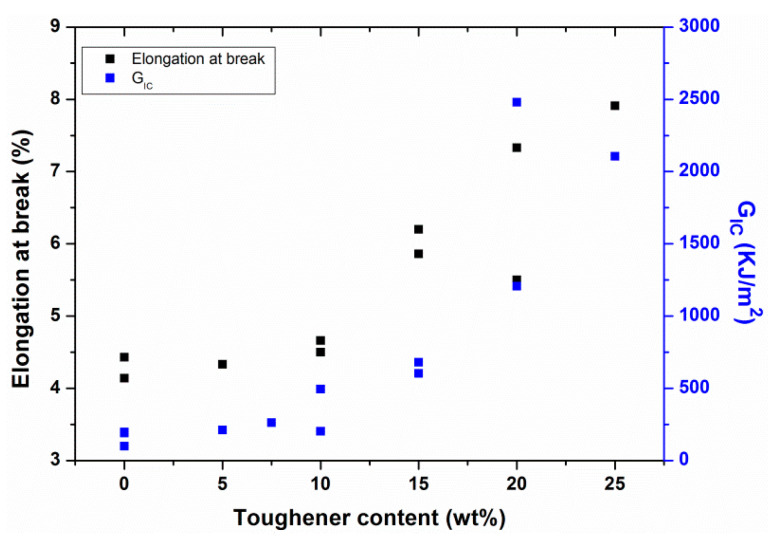
Elongation at break and G_IC_ values as a function of toughener content for BADCy/PEI modified system taken from refs. [32,33,82].

In summary, the concept of (semi-)IPN formation proved to be a useful alternative for the toughening of epoxy resin for aerospace applications. In addition to the enhancement in mechanical properties (i.e., fracture toughness), the development of (semi-)IPN structure in epoxy based adhesives can also facilitate the joining of different composite parts with better mechanical properties, which is a crucial requirement for the aerospace industry. Furthermore, PEI appears to be a potential candidate for the toughening of epoxy resin due to its higher *T*_g_, higher strength and rigidity at elevated temperatures, good compatibility with epoxy, etc. The incorporation of PEI in epoxy resin usually results in the formation of a semi-IPN structure, which eventually provides the improved mechanical characteristics, i.e., fracture toughness of toughened epoxy system. For example, elongation at break and fracture toughness of BADCy/PEI system are plotted as a function of toughener content (i.e., PEI) (Figure 11). Three regions can be identified from the graph: (i) below 10 wt % of toughener content where a small increase in both properties is observed; (ii) from 10 to 20 wt % of toughener content where an almost a linear increase in mechanical properties (e.g., elongation at break and fracture toughness) is evident; (iii) above 20 wt % where a slight decrease or a plateau behavior in the properties is observed. The slight increase in mechanical properties of epoxy system below 10 wt % of toughener content is due to the incorporation of a ductile material and the formation of particulate morphology. The linear increase in fracture toughness/elongation at break of epoxy resin at 10–20 wt % toughener content is attributed to the formation of IPN structure. The plateau behavior or a decrease in mechanical properties of epoxy resin above 20 wt % of PEI is associated with the fact that there are no further interactions between the epoxy and toughener and also with the coalescence of toughener phase morphologhy. Furthermore, apart from pure epoxy modification with PEI, toughened polymer composites have also been developed and analyzed particularly for aerospace applications. However, further research is still needed to efficiently utilize the thermoplastic modification approach for aerospace grade polymeric composites, in addition to the epoxy resin. 

## 5. Conclusions

Epoxy resins are attractive matrix systems for fibre reinforced polymer composites in the aerospace sector due to excellent properties such as ease of processing, low cost, superior mechanical, thermal and electrical properties. However, the brittleness and low elongation after cure of pure epoxy system restricts its potential. In literature, several strategies have been adopted to solve this problem. Amongst all those methodologies, formation of interpenetrating polymer network (IPN), by incorporating either thermoplastic or thermoset in brittle epoxy, is an attractive approach to improve the fracture toughness of modified system. This methodology usually results in better mechanical properties (e.g., fracture toughness) of the modified epoxy system. Toughener content, chain mobility, chemical functionalization and morphology seem to be the main drivers affecting the fracture toughness of such systems.

Several parameters have been investigated in literature to tune the mechanical properties (i.e., fracture toughness and impact strength) of the toughened epoxy system. For instance, the toughener content (either thermoplastic or thermoset) has been observed to be a crucial parameter in controlling the mechanical properties of resultant epoxy system. The increase in toughener content typically leads toward the gradual increase in fracture toughness of epoxy resin, which is attributed to the formation of IPN structure. However, after a certain level of concentration of toughener, the fracture toughness of modified epoxy system approaches a plateau behavior, which is associated with the fact that there are no further interactions between the epoxy and toughener. In the case of impact and tensile strength, after certain concentration of toughener, a decreasing trend is usually observed which is linked with the formation of toughener rich domains, which eventually inhibits the development of IPN structure. However, this decrease in impact strength of epoxy system can be tackled by playing with the branching and structure of the thermosetting toughener. In addition to the toughener content, other parameters have also been observed to affect the mechanical properties of toughened epoxy resin such as molecular weight of toughener components, different curing agents, etc. Hence, the final mechanical properties including fracture toughness of the modified epoxy system can be finely tuned by controlling the phase morphology and/or creating the IPN structure.

Moreover, the concept of (semi-)IPN formation using PEI proved to be an interesting alternative for the toughening of epoxy resin for aerospace applications, due to its higher *T*_g_, higher strength and rigidity at elevated temperatures, good compatibility with epoxy, etc. This IPN formation approach can further facilitate the joining of different composite parts with superior mechanical properties, which is an essential process in the aerospace industry. In addition to the pure epoxy modification, the preparation of polymer composites from toughened epoxy resin was also performed. In the case of modified epoxy system based on thermosetting toughener, polymer composites display better mechanical properties (e.g., fracture toughness) as compared to the un-modified epoxy while the thermoplastic toughening approach usually reduces the fracture toughness of the toughened polymer composite. Therefore, further research is still needed to efficiently utilize the thermoplastic modification approach to prepare the aerospace grade polymeric composites. For instance, the formation of (semi-) IPN using thermoplastic multilayers (i.e., thin films) for epoxy toughening and the utilization of this toughened epoxy system for producing aerospace grade polymeric composites would be an interesting research area for future work.

## Figures and Tables

**Table 1:** Brief summary of different interpenetrating polymer network (IPN) based epoxy systems with thermoplastics toughener.

Epoxy	Toughener	Toughener Content	Morphology	Investigated Properties	Key Findings	Ref.
DGEBA/DDS/BMI	PPO	10–25 wt %	Micron-sized fibrous structure	Elemental analysis Thermal properties Fracture toughness Impact strength	The epoxy modified with PPO displayed 63% higher fracture toughness and 30% higher tensile strength as compared to the neat system at room temperature, which was attributed to the reinforcing and toughening functions of aligned PPO fibres, in addition to the IPN. The fracture toughness increased (from 50 to 63%) by increasing the content of PPO from 10 to 25 wt %.	[44]
DGEBA/NMA/MHHPA	FCBHBP	10–30 wt %	Ridge morphology	Elemental analysis Electrical properties Thermal properties Bending strength Impact strength	By adding 20 wt % of toughener, an increase of 122% and 184% was observed in bending strength and impact strength, respectively. This increase was linked to the formation of IPN between flexible chain of toughener and epoxy molecular chain.	[10]
DGEBA/DDS	MABS	4.76–16.6 wt %	Co-continuous morphology	Elemental analysis Impact strength Tensile strength	The results showed an increase in tensile strength and impact strength of neat epoxy of about 25% and 72%, respectively, by adding MABS.	[39]
DGEBA/DDS	PEEK-PR	4.76–13 wt %	Inverted phase morphology	Elemental analysis Thermal properties Tensile strength Impact strength Fracture toughness Rheology	In this study, an increase of 33% in fracture toughness at room temperature was reported by adding PEEK-PR into the epoxy system. The results showed an increasing behavior of fracture toughness as a function of increasing toughener content.	[38]
Bisphenol A type epoxy	Polymer prepared from PPG, PEG and MDI	10–50 wt %	Two-phase structure	Elemental analysis Thermal properties Bonding strength Failure strain	The results showed an increase in bonding strength of epoxy till 20 wt % of toughener and after that a decline in properties was observed, which was linked with the change in morphology.	[12]
DGEBA/TMHDA	9G	25–75 wt %	Discrete morphology	Elemental analysis Thermal properties Tensile strength ∙ Elongation at break	The maximum tensile strength and elongation at break were obtained for the modified epoxy system with equal content of epoxy and toughener (i.e., 50/50).	[52]
CDGE/IPD	Poly(EDBz)	20–80 wt %	Phase inversion	Elemental analysis Thermal properties Tensile strength Compressive strength Impact strength	This study reported an increase in storage moduli and other mechanical properties of epoxy as a function of increasing Poly(EDBz) content.	[53]
DGEBA/DMPA	Polysulfide prepared in-situ from thiol-ene system	10–40 mol %	Micro-heterogeneous structure	Elemental analysis Thermal properties Fracture toughness Impact strength	The results revealed the enhancement in the mechanical properties of epoxy system as a function of increasing content of polysulfide due to its flexible nature.	[43]
Bisphenol A type epoxy	STU	5–20 wt %	-	Impact strength Wear resistance	The impact strength of modified epoxy increased till 10 wt % of STU and decreased after further increase in STU content. However, the results showed a continuous increase in wear resistance as a function of increasing STU content.	[54]
DGEBA/MOCA	PUP	30–70 wt %	Dispersed or bi-continuous phases	Tensile strength Impact strength Wear resistance	The best tensile strength of modified epoxy was obtained by adding 30 wt % of PUP and decreased by further addition of PUP. The impact strength, on the other hand, increased by increasing PUP content. The wear performance of epoxy systems with 30 and 50 wt % of PUP was better than the next epoxy and PUP.	[50]
Bisphenol A type epoxy/G1-G3	RTA	7.6–78 wt %	Sea-island structure	Elemental analysis Thermal properties Tensile strength Impact strength Fracture toughness	By increasing the toughener content, tensile strength decreased while the elongation at break increased, which was attributed to the transformation of morphology from sea-island structure to IPN.	[55]
DGEBA/H-957/DADPE/DADPS	SiMPU	4.8–28.6 wt %	-	Fracture toughness Impact strength	The impact strength and fracture toughness at room temperature of modified epoxy system increased gradually till 16.7 wt % of SiMPU with highest values of 25 J/m and 3 MPa·m^1/2^, respectively, at this concentration.	[11]
Bisphenol A type epoxy/Polyamide	PUP	9–23 wt %	Two-phase structure	Elemental analysis Compressive strength Impact strength Thermal properties	The results showed an increase in impact and compressive strength as a function of increasing PUP content till 16.7 wt % and a decrease afterwards. The optimum mechanical properties of modified epoxy were obtained with 16.7 wt % of PUP including compressive strength of 184.8 MPa and impact strength of 76.6 kJ/m^2^.	[51]
DGEBA/DDM	PBHDDP	0.99–11.11 wt %	Homogeneous distribution (i.e., no phase separation)	Thermal properties Tensile strength Impact strength Fracture toughness ∙ Flame retardancy	Improvement in mechanical properties of epoxy by incorporating toughener without losing useful properties such as clarity and *T*_g_. In this study, 150% increase in toughness of epoxy at room temperature was reported. This enhancement in properties was observed till 2.44 wt % of toughener and after that a linear decrease in properties was evident.	[24]
DGEBA/D-230	Co-polyimide	30–50 wt %	Micro heterogeneous morphology	Thermal properties Tensile strength	Improvement in mechanical properties and thermal stability of epoxy system by increasing the thermoplastic content.	[40]
TGAP/DDS	Poly(p-BAB/SPB), Poly(m-BAB/SPB)	5–15 wt %	Simultaneous existence of co-continuous and spherical domains	Viscosity Thermal properties Fracture toughness	The results showed that only 5 wt % of poly(p-BAB/SPB) was required to obtain the fracture toughness higher than the toughness of epoxy system modified with 20 wt % of PES.	[35]
TGAP/DDS	PES	4.76–16.67 wt %	Co-continuous domains	Thermal properties Fracture toughness Impact strength Tensile strength	An increase of 11% in fracture toughness at room temperature of neat epoxy was reported by incorporating PES.	[36]
BADCy	Polyimide	3–20 wt %	Co-continuous domains	impact strength of semi-IPN was 47–320% greater than that of polycyanurate. Thermal properties Impact strength Tensile strength Flexural strength	The impact strength of the thermoplastic/epoxy blend was observed to be higher (47–320%) than the neat epoxy (polycyanurate), due to the formation of semi-IPN.	[42]

**Table 2:** Brief summary of different IPN based systems investigated in literature for epoxy modification as a function of different parameters.

Epoxy	Toughener	Morphology	Objective	Investigated Properties	Key Findings	Ref.
DGEBA/INN	Polyurethane obtained from TDI and Desmophen 1200	No phase separation	To investigate the effect of isocyanate content	Elemental analysis Tensile strength Impact strength	The better mechanical properties of the modified epoxy system was achieved by using toughener with higher isocyanate content. The impact strength was maximally enhanced by adding 20% of polyurethane with highest isocyanate index while the addition of 15% of the toughener was enough to obtain the best tensile strength and elongation at break.	[58]
DGEBA/TETA	Polyurethane derived from PET waste by glycolysis	Homogeneous morphology	To analyze the influence of molecular weight ofpolyethylene glycol (600–1500 g·mol^−1^)	Fracture toughness Tensile strength Impact strength	The results showed the maximum increase of 61% and 212% in tensile strength and impact strength, respectively, by adding 10 wt % of polyurethane prepared with the glycol having molecular weight of 1000. Moreover, the addition of toughener resulted in 45% and 184% increase in mode I fracture toughness and fracture energy, respectively, as compared to the neat epoxy system at room temperature.	[57]
DGEBA/modified amine	PUP	Sea-island structure	To understand the influence of different gravity accelerations of 0, 1, and 2 *g*	Elemental analysis Bending stress Thermal properties	The bending stress and coefficient of thermal expansion (CTE) of modified epoxy showed a decrease with the increasing acceleration of gravity which was attributed to the increase in diameter and decease in number of dispersed phase.	[59]
DGEBA/PAMAM	HBP-PU	Two-phase morphology	To study the impact of different generations (G) of HBPs (G1–G4)	Elemental analysis Thermal properties Flexural strength Impact strength	The results showed an increase in the impact strength of modified epoxy till generation G3 and a decrease for G4, which was attributed to the formation of bigger particles. However, the flexural modulus and strength linearly increased as a function of generation because of the enhancement in the rigidity of the system. The HBP-PU based epoxy samples displayed higher toughness as compared to the next epoxy and linear-PU based epoxy samples. While the thermal stability and flexural characteristics of HBP-PU based epoxy system were lower than the neat epoxy which was linked with the presence of flexible linkages and reduction in cross-linked density of epoxy.	[13]
Bisphenol A type epoxy	RTA	Sea-island structure	To examine the effect of different curing agents (G1–G3)	Elemental analysis Thermal properties Tensile strength Impact strength Fracture toughness	The results showed that the mixtures having G1 and G2 curing agent satisfied the technical index requirements of steel deck pavement. Moreover, the fatigue life of epoxy mixture cured with G1 was quite longer than the epoxy asphalt mixture.	[55]
DGEBA	SiMPU	-	To assess the influence of different curing agents (H-957, DADPE and DADPS)	Fracture toughness Impact strength	The values of impact strength and fracture toughness (at room temperature) of epoxy system, cured by using H-957, were higher than the epoxy systems cured with other two hardeners (DADPE and DADPS). The results showed an increase of 216% in fracture toughness by curing the epoxy with H-957 along with 16.7 wt % of SiMPU, as compared to the neat epoxy.	[11]

**Table 3:** Brief summary of different IPN based systems investigated in literature for epoxy modification using thermosets.

Epoxy	Toughener	Morphology	Investigated Properties	Key Findings	Ref.
DGEBA/NMA	PDCPD	Co-continuous phase-separated structures	Bond strength Tensile strength Fracture toughness	The blended epoxy system displayed better mechanical properties than the individual components. The results showed an increase in energy dissipation mechanisms (i.e., fracture toughness) by increasing PDCPD content in epoxy. The epoxy system having 70% of PDCPD exhibited bond strength of ~35% and ~125% higher than the neat epoxy and neat PDCPD, respectively.	[69,70]
ECH	BPAEDA	-	Elemental analysis Thermal properties Elastic modulus Failure strain	The nano-indentation results showed the elastic modulus of 16.7–715 MPa for modified epoxy system.	[65]
BADCy	FETI	-	Elemental analysis Thermal properties Tensile strength Impact strength Rheology	The superior thermal and mechanical properties of blended epoxy were observed with the *T*_g_ of 303–312 °C, tensile strength of 87–95 MPa and impact strength of 27–37 kJ·m^−2^. These enhancements were attributed to the presence of high fluorine content, low crosslinked density, high chain rigidity, and strong intra- and intermolecular interactions.	[66]
DGEBA/IPD	UP and VE CF and GF	Phase separation	Impact strength Tensile strength Thermal properties Fracture toughness	The hybridization of epoxy with VE resulted in enhanced toughness, damping and energy absorbing properties as compared to the neat individual components at room temperature. In case of composites, the same hybrid epoxy (EP/VE) showed better mechanical properties such as flexural strength and inter-laminar properties.	[68]
DGEBA/DDM	HTTE	Sea-island morphology	Elemental analysis Thermal properties Fracture toughness Impact strength	In this study, the fracture toughness was observed to increase with the increasing content of HTTE at room temperature. The thermal stability of hybrid epoxy decreased with the increasing HTTE content but increased by changing the generation of HTTE from 1 to 3. This increase in thermal stability of hybrid epoxy was linked with the increase in molar mass, intermolecular interactions of HTTE/epoxy and crosslink density.	[64]
Bio-based epoxy resin	ESO and PFA	Non-homogenous network	Elemental analysis Thermal properties Tensile strength Impact strength	The results showed that the incorporation of ESO and PFA in epoxy system resulted in 76.6% higher impact strength, as compared to the neat epoxy, which was associated to the addition of flexible ESO chains. However, the tensile strength and glass transition temperature decreased by adding ESO and PFA in epoxy.	[67]
DGEBS/DDS	TGDDM	Homogeneous morphology	Thermal properties Fracture toughness Elastic modulus	The incorporation of TGDDM into the epoxy/copolymer system resulted in increased glass transition temperature without affecting the fracture toughness.	[71]
UVR/MHHPA	TMPTMA, OMMTs and SLFs	Two-phase morphology	Thermal properties Bending strength Impact strength	The mechanical and thermal properties of epoxy blend were enhanced by adding the fillers (OMMTs and SLFs). The optimum toughening-strengthening properties were obtained by incorporating 0.5 wt % of SLFs and 4.8 wt % of OMMTs in epoxy blend.	[63]

**Table 4:** Mechanical properties of different epoxy/poly(ether imide) (PEI) systems investigated in literature at room temperature.

Sample	Molecular Weight of PEI, *M*_n_ (g/mol)	Tensile Strength (MPa)	Tensile Modulus (GPa)	Elongation at Break (%)	K_IC_ (MPa·m^1/2^)	G_IC_ (kJ/m^2^)	Ref.
BADCy		63.7	2.27	4.14	0.62	0.100	[33]
BADCy/PEI = 90/10	12,000	75.6	2.38	4.66	1.35	0.495	[33]
BADCy/PEI = 85/15	12,000	84.7	2.39	5.86	1.6	0.680	[33]
BADCy/PEI = 80/20	12,000	73.4	2.00	5.50	3.1	2.480	[33]
BADCy		~84.2	~2.60	~4.43	-	~0.190	[82]
BADCy/PEI = 95/5	18,000	~82.1	~2.57	~4.33	-	~0.211	[82]
BADCy/PEI = 90/10	18,000	~83.4	~2.54	~4.50	-	~0.202	[82]
BADCy/PEI = 85/15	18,000	~99.2	~2.53	~6.20	-	~0.603	[82]
BADCy/PEI = 80/20	18,000	~108.4	~2.54	~7.33	-	~1.207	[82]
BADCy/PEI = 75/25	18,000	~110.9	~2.55	~7.91	-	~2.105	[82]
HPT		-	-	-	~0.60	-	[78]
HPT/PEI = 95/5	20,000	-	-	-	~1.18	-	[78]
HPT/PEI = 90/10	20,000	-	-	-	~1.22	-	[78]
HPT/PEI = 85/15	20,000	-	-	-	~1.35	-	[78]
BADCy		-	-	-	-	~0.196	[32]
BADCy/PEI = 92.5/7.5	18,000	-	-	-	-	~0.261	[32]
DGEBA/PEI = 87/13	19,000	~49.3	~0.67	~7.1	-	-	[83]
TGDDM + DGEAC		62.0	2.6	3.2	-	-	[84]
TGDDM + DGEAC/PEI = 85/15	23,825	80.9	2.6	3.5	-	-	[84]
TGDDM + DGEAC/PEI = 80/20	23,825	88.8	2.6	4.7	-	-	[84]
TGDDM + DGEAC/PEI = 85/15	25,405	87.3	2.7	4.3	-	-	[84]
TGDDM + DGEAC/PEI = 80/20	25,405	88.8	2.6	4.7	-	-	[84]
TGDDM + DGEAC/PEI = 85/15	21,175	77.0	2.6	3.9	-	-	[84]
TGDDM + DGEAC/PEI = 80/20	21,175	81.6	2.6	4.2	-	-	[84]
TGDDM + DGEAC/PEI = 85/15	20,983	89.2	2.7	4.4	-	-	[84]
TGDDM + DGEAC/PEI = 80/20	20,983	86.6	2.7	4.4	-	-	[84]

## Appendix A

List of abbreviations

DGEBA	Diglycidyl ether of biphenol A
DDM	4,4′-Diaminodiphenyl methane
PBHDDP	poly[4,4′-bis(6-hydroxyhexyloxy)biphenyl 9,10-dihydro-10-[2,3-di(hydroxycarbonyl) propyl]-phosphaphenanthrene-10-oxide]
TGAP	Triglycidyl p-aminophenol
DDS	4,4′-diaminodiphenyl sulfone
Poly(p-BAB/PBP)	Poly(p-1,4-bis(azidomethyl)benzene/4,4′-bis(2-propynyloxy)benzophenone)
Poly(p-BAB/SPB)	Poly(p-1,4-bis(azidomethyl)benzene/4,4′-sulfonylbis(propynyloxy)benzene)
Poly(m-BAB/SPB)	Poly(p-1,3-bis(azidomethyl)benzene/4,4′-sulfonylbis(propynyloxy)benzene)
PES	Polyethersulfone
D-230	Poly(propylene glycol) bis (2-aminopropyl ether)
BADCy	bisphenol A dicyanate
BMI	4,4′-bismaleimidodiphenylmethane
PPO	polyphenylene ether
MHHPA	Methylhexahydrophthalic anhydride
FCBHBP	Flexible chain blocking hyperbranched polyester
MABS	Methyl methacrylate acrylonitrile butadiene styrene
PEEK-PR	PEEK oligomer with terminal propargyl groups
PEEK-TOH	Hydroxyl terminated PEEK
PPG	Polypropylene glycol
PEG	Polyethylene glycol
MDI	4,4′-Diphenylmethane diisocyanate
TMHDA	2,2,4-trimethylhexane-1,6-diamine
9G	Poly(ethylene glycol dimethacrylate)
CDGE	Cardanol diglycidyl ether
IPD	Isophorone diamine
Poly(EDBz)	Polybenzoxazine
DMPA	2,2-dimethoxy-2-phenylacetophenone
INN	Triethylenetetramine
TDI	Toluene diisocyanate
TETA	Triethylene tetramine
PUP	Polyurethane prepolymer
PAMAM	Polyamidoamine
HBP-PU	Hyperbranched polyester-Polyurethane
RTA	Polyurethane active toughening agent
STU	Silane terminated urethane
MOCA	3,3′-dichloro-4,4′-diamino diphenyl methane
H-957	Ciba–Geigy two-pack hardener
DADPE	Diaminodiphenyl ether
DADPS	Diaminodiphenyl sulfone
SiMPU	Hydroxy-terminated silicon-modified polyurethane
NMA	Nadic methyl anhydride
PDCPD	Polydicyclopentadiene
UVR	3,4-epoxycyclohexylmethyl 3′,4′-epoxycyclohexane carboxylate
TMPTMA	Trimethylol-1,1,1-propane trimethacrylate
OMMTs	Organic montmorillonties
SLFs	Scrap leather fibers
ECH	3,4-epoxycyclohexylmethyl 3,4-epoxycyclohexanecarboxylate
BPAEDA	Bisphenol A ethoxylate dimethacrylate
FETI	Fluorinated ethynyl-terminated imide oligomers
UP	Orthophtaleic acid based resin
CF	Carbon fibre
GF	Glass fibre
HTTE	Hyperbranched poly(trimellitic anhydride-triethylene glycol) ester epoxy
ESO	Epoxidized soybean oil
PFA	Poly(furfuryl alcohol)
DGEBS	Diglycidyl ether of bisphenol S
TGDDM	Tetraglycidyl-4,4-diaminodiphenylmethane
HPT	*N*,*N*,*N*′,*N*′-tetraglycidyl-α,α′-bis-(4-aminophenyl)-*p*-diisopropylbenzene
DGEAC	Diglycidylester of aliphatic cyclo epoxy
HBP	Hyperbranched polyesters
HMDI	Hexamethylene diisocyanate
PEN	Poly(arylene ether nitrile)
BA-ph	Phthalonitrile containing benzoxazine
PMSQ	Polymethylsilsesquioxane
